# Altered Hemispheric Asymmetry of Functional Hierarchy in Schizophrenia

**DOI:** 10.3390/brainsci15030313

**Published:** 2025-03-16

**Authors:** Yi Zhen, Hongwei Zheng, Yi Zheng, Zhiming Zheng, Yaqian Yang, Shaoting Tang

**Affiliations:** 1 School of Mathematical Sciences, Beihang University, Beijing 100191, China; 2 Key Laboratory of Mathematics, Informatics and Behavioral Semantics, Beihang University, Beijing 100191, China; 3 Beijing Academy of Blockchain and Edge Computing, Beijing 100085, China; 4 Institute of Artificial Intelligence, Beihang University, Beijing 100191, China; 5 Hangzhou International Innovation Institute, Beihang University, Hangzhou 311115, China; 6 Institute of Medical Artificial Intelligence, Binzhou Medical University, Yantai 264003, China; 7 Zhongguancun Laboratory, Beijing 100094, China; 8 Beijing Advanced Innovation Center for Future Blockchain and Privacy Computing, Beihang University, Beijing 100191, China; 9 State Key Laboratory of Complex & Critical Software Environment, Beihang University, Beijing 100191, China

**Keywords:** hemispheric asymmetry, functional gradient, schizophrenia, functional connectome, resting-state fMRI

## Abstract

Background/Objectives: Schizophrenia is a severe psychiatric disorder characterized by deficits in perception and advanced cognitive functions. Prior studies have reported abnormal lateralization in cortical morphology and functional connectivity in schizophrenia. However, it remains unclear whether schizophrenia affects hemispheric asymmetry in the hierarchical organization of functional connectome. Methods: Here, we apply a gradient mapping framework to the hemispheric functional connectome to estimate the first three gradients, which characterize unimodal-to-transmodal, visual-to-somatomotor, and somatomotor/default mode-to-multiple demand hierarchy axes. We then assess between-group differences in intra- and inter-hemispheric asymmetries of these three functional gradients. Results: We find that, compared to healthy controls, patients with schizophrenia exhibit significantly altered hemispheric asymmetry in functional gradient across multiple networks, including the dorsal attention, ventral attention, visual, and control networks. Region-level analyses further reveal that patients with schizophrenia show significantly abnormal hemispheric gradient asymmetries in several cortical regions in the dorsal prefrontal gyrus, medial superior frontal gyrus, and somatomotor areas. Lastly, we find that hemispheric asymmetries in functional gradients can differentiate between patients and healthy controls and predict the severity of positive symptoms in schizophrenia. Conclusions: Collectively, these findings suggest that schizophrenia is associated with altered hemispheric asymmetry in functional hierarchy, providing novel perspectives for understanding the atypical brain lateralization in schizophrenia.

## 1. Introduction

Schizophrenia (SZ) is a debilitating psychiatric disorder with a prevalence of roughly one percent of the world’s population [1], commonly manifesting in hallucinations, delusions, disturbances in thought and behavior, and cognitive impairments [2,3,4]. A substantial body of neuroimaging research has identified abnormalities in both structural and functional connectome [5,6], supporting that SZ can be characterized as a disconnection syndrome [7,8]. These connectome abnormalities extend from primary unimodal regions (e.g., somatomotor network) to transmodal regions (e.g., default mode network) [9,10], in parallel with sensory deficits and impairments in high-order cognitive processing [11,12]. Through a connectome perspective, these studies provide critical insights into the intricate pathophysiology of SZ.

Apart from abnormal connectivity, convergent evidence has revealed that SZ is associated with abnormal lateralization in brain morphology [13,14], structural and functional connectomes [15,16], indicating disrupted hemispheric specialization. Hemispheric specialization, as a fundamental organizational principle of the human brain [17], serves as a hallmark of successful neurodevelopment [18,19] and relates to lateralized cognitive functions [20,21]. Left-hemisphere dominance is considered to be relevant to language and reasoning capacities [22,23], whereas right-hemisphere dominance is associated with emotion processing and visuospatial attention [24,25]. Hemispheric specialization is regarded as a critical characteristic for reducing redundancy and enhancing efficiency, thus facilitating flexible and parallel information processing to meet complex cognitive demands [26,27]. Ample research has reported that, compared to healthy controls, patients with SZ exhibited abnormal hemispheric lateralization in functional activations [28,29,30], the variability of fMRI signals [31], and functional connectivity [15,19,32,33,34]. Despite some inconsistencies in these studies arising from different functional measures and analytical samples, some brain regions, such as language-related areas [28,29], frontoparietal regions [33,34], and temporal lobes [31,32], are frequently reported to exhibit abnormalities in functional lateralization in patients with SZ. The largest case-control study from the ENIGMA consortium has demonstrated altered hemispheric asymmetry in pallidum volume and cortical thickness of the middle temporal gyrus and rostral anterior cingulate in patients with SZ [14]. Altered structural and functional asymmetries in SZ are associated with psychotic symptoms and may reflect aberrant specialization of cognitive processes supporting language and emotion [14,16,30,32]. While abnormal lateralization in functional measures, such as functional activation and functional connectivity, has been well documented in patients with SZ, it remains unclear whether SZ is associated with altered asymmetry in the functional hierarchy, an essential aspect of functional organization that enables information encoding and integration from sensation to cognition [35,36].

By mapping macroscale functional connectome into a low-dimensional embedding space, the recently developed functional gradient method provides a compelling framework to elucidate the functional hierarchical architecture of interactions among distributed brain regions [37]. This framework embeds regions with similar functional connectivity profiles into proximate positions along low-dimensional axes, ultimately yielding a series of continuous spatial arrangements, known as functional gradients. Previous studies have demonstrated that the spatial variation of functional gradients offers information regarding the integration and segregation of connectivity profiles across distributed regions [38,39]. For example, the principal gradient describes a macroscale hierarchical organization where sensory/motor regions and default mode regions are positioned at opposite ends, in accordance with a progressively abstract functional spectrum that transitions from specialized to integrated information processing [37]. Extensive research has employed functional gradients to characterize changes in the hierarchical architecture of functional connectome across development and aging [40,41], during various cognitive tasks [42,43], and in neuropsychiatric disorders [36,44]. Specially, prior studies have reported that patients with SZ show a compression of both the principal cortical and cerebellar functional gradients [45,46], as well as an expansion of the unimodal-to-transmodal thalamic functional gradients [47]. Recent studies have demonstrated the hemispheric asymmetry of functional gradients, which is heritable, phylogenetically conserved, and sex-related [48,49]. However. whether the hemispheric lateralization of functional gradients is influenced by SZ remains unknown.

We concentrated on the asymmetry of functional hierarchy, which has been shown to provide critical insights into brain lateralization [48] and the atypical brain lateralization observed in autism [50] and major depressive disorder [51]. We aimed to investigate whether SZ alters the hemispheric asymmetry of functional hierarchical organization, enhancing our understanding of abnormal brain lateralization in patients with SZ. To achieve this, we utilized the diffusion mapping method to estimate the first three hemispheric functional gradients in both healthy controls and patients. We focused on hemispheric asymmetries in the hierarchical organization of both intra- and inter-hemispheric connectivity. The intra-hemispheric and inter-hemispheric connectivity organizations are thought to offer complementary insights: the former is believed to indicate corpus callosum inhibition and hemispheric specialization; the latter is considered to support signal transmission and information integration between two hemispheres [48,52]. We conducted case-control comparisons of hemispheric gradient asymmetries at both the network and regional levels. We hypothesized that patients with SZ would exhibit significantly altered intra-hemispheric and inter-hemispheric gradient asymmetries compared to healthy controls. Finally, we adopted predictive models to examine whether the hemispheric gradient asymmetry could act as a marker for precisely classifying patients and healthy individuals, and whether it could predict the clinical symptoms of SZ.

## 2. Materials and Methods

### 2.1. Participants and Data Acquisition

We harmonized resting-state fMRI data from eight publicly available datasets [53,54,55,56,57,58,59]. Due to the low proportion of left-handed participants in these datasets, we retained only right-handed participants to ensure that our analysis of brain lateralization would not be confounded by handedness. Following rigorous image quality control (see below and Supplementary Methods), a total of 479 participants, comprising 270 healthy controls and 209 patients with schizophrenia (SZ), were included in subsequent analyses. Informed consent was obtained from all participants, and data collections were approved by the institutional review boards corresponding to data acquisition sites. More details regarding participant diagnosis and MRI data acquisition were provided in Supplementary Methods.

### 2.2. Data Preprocessing

Structural and functional MRI data were preprocessed using fMRIPrep 20.2.3 [60]. To summarize, the preprocessing procedures for structural data included correcting intensity non-uniformity, skull stripping, segmenting brain tissues, reconstructing cortical surface, and normalization to standard MNI spaces. For functional data, the preprocessing steps comprised correcting for head motion, slice-timing correction, the alignment between functional and structural data, and resampling to standard MNI spaces. For more details on fMRIPrep preprocessing, refer to Supplementary Methods. The preprocessed functional data were further denoised [61], which included removal of the linear trend, band-pass temporal filtering (0.01–0.08 Hz), and nuisance regression of 24 head motion parameters [62,63], mean cerebrospinal fluid (CSF) signals, and mean white matter signals. The nuisance regression was performed orthogonally to temporal filtering [64]. MRI data from participants were excluded from this study if any of the following criteria were met: (1) inadequate brain extraction, (2) poor structural image segmentation, (3) bad registration between functional and structural data, or (4) excessive motion in functional data (mean framewise displacement (FD) [65] > 0.35 mm or maximum FD > 3 mm). Finally, we utilized a 400-region homotopic atlas [66] to parcellate the functional data and extracted regional average fMRI time series.

### 2.3. Hemispheric Functional Gradients

We constructed the individual functional connectivity (FC) matrix by calculating the Pearson correlation of mean time series between regions, and then applied Fisher’s Z transformation to convert these correlations into z-values. Consistent with previous studies [48,50,51], we divided the FC matrix into four distinct parts: FC within the left hemisphere (LL connectome), FC within the right hemisphere (RR connectome), FC from the left to the right hemisphere (LR connectome), and FC from the right to the left hemisphere (RL connectome). This procedure yielded four hemispheric FC matrices for each participant, comprising two intra-hemispheric FC matrices and two inter-hemispheric FC matrices. For each participant, we estimated functional gradients of each hemispheric FC matrix using the BrainSpace toolbox [67]. Consistent with prior studies [37,44,50,51], we applied a threshold to each column of the hemispheric FC matrix, retaining only the top 10% of the strongest functional connections. We constructed an affinity matrix for each thresholded hemispheric matrix by computing the normalized angle similarity coefficient between regional FC profiles [50,51]. We subsequently performed a nonlinear diffusion map embedding on each affinity matrix to extract multiple continuous components (namely functional gradients) that were ranked in descending order according to the explained variance [37,44]. This process considers the affinity matrix as a graph and transforms the high-dimensional connectivity matrix into its low-dimensional embedding representation. Along low-dimensional axes, nodes with numerous suprathreshold connections or a few very strong connections are positioned closer together, while nodes with little or no interconnectivity are situated farther apart [42]. The diffusion map embedding algorithm is controlled by two parameters, i.e., *t* and α. In line with prior research [37,44], we set t=0 and α=0.5 to maintain global relationships between nodes within the embedded space, which is also the default parameter setting of the BrainSpace software. To ensure comparability of hemispheric functional gradients across participants, we generated two hemispheric gradient templates based on resting-state fMRI data from the Human Connectome Project (HCP) [68], which has been widely employed for constructing gradient templates [50,69]. For convenience, we selected fMRI data from the “unrelated 100 subjects” dataset in the HCP database. We estimated an intra-hemispheric gradient template based on the group-average intra-hemispheric FC matrix, which was generated by averaging all intra-hemispheric FC matrices (LL and RR connectomes). Similarly, we estimated an inter-hemispheric gradient template based on the group-average inter-hemispheric FC matrix, which was derived by averaging all inter-hemispheric FC matrices (LR and RL connectomes). Using Procrustes rotation [70], we aligned two intra-hemispheric gradients of each individual to the intra-hemispheric gradient template, and aligned two inter-hemispheric gradients of each individual to the inter-hemispheric gradient template.

### 2.4. Asymmetry Index

In line with prior studies [48,50,51], we calculated an asymmetry index (AI) to evaluate the hemispheric asymmetry of functional gradients. For intra-hemispheric asymmetry, AI was computed by left intra-hemispheric gradient scores minus right intra-hemispheric gradient scores (LL–RR). Similarly, AI for inter-hemispheric asymmetry was calculated as the difference between left-to-right and right-to-left inter-hemispheric gradient scores (LR–RL). A positive AI value indicates leftward asymmetry, meaning that the region in the left hemisphere shows a higher gradient score compared to its homologous region in the right hemisphere.

### 2.5. Between-Group Comparison of Gradient Asymmetries

We focused on the first three functional gradients to assess between-group differences in hemispheric gradient asymmetries (AI). We performed between-group comparisons at both network-level and region-level. Considering that the fMRI data were collected from multiple different sites, we first corrected for multi-site effects using the NeuroComBat harmonization method [71] prior to conducting comparisons, with group, age, sex, and mean FD as covariates. For network-level analyses, we averaged regional hemispheric gradient scores according to Yeo’s seven functional networks [72], which encompassed the visual, somatomotor, dorsal attention, ventral attention, limbic, control, and default mode networks. We computed network-level intra-hemispheric or inter-hemispheric AI values by the difference between two hemispheric gradient scores. We then applied a linear model to evaluate the differences in AI values between healthy controls and patients with SZ. Multiple comparison corrections were conducted using the FDR method, and the significance threshold was set at corrected p<0.05. For region-level analyses, we utilized linear models to compare between-group differences in regional AI values for each gradient, with statistical significance set at FDR-corrected p<0.05. For all between-group comparisons at both the network and regional levels, age, sex, and mean FD were included as covariates. All surface visualizations of regional analyses were generated using the Python (version: 3.10.12) packages BrainSpace (version: 0.1.10) [67] and Surfplot (version: 0.2.0) [73].

### 2.6. Prediction

We selected AI values that passed the significance test in the network-level and region-level analysis as features. These features effectively reflect the SZ-related alterations in the lateralization of functional connectome organization across three gradient axes. We first trained a logistic regression model with L2 regularization to investigate whether these selected features could identify patients and healthy controls. We performed a five-fold nested cross-validation to establish models and assess their performance. We randomly divided the individuals into training and test sets in a 4:1 ratio and repeated this process 100 times. For each training set, we conducted another five-fold cross-validation to choose the optimal hyperparameter (the inverse of the regularization strength). The model performance was assessed on the test set using classification accuracy and the area under the receiver operating characteristic (ROC) curve (AUC). Subsequently, we used a ridge regression model to examine whether these features could predict the positive and negative syndrome scale (PANSS) scores of patients. We applied a five-fold nested cross-validation strategy, randomly dividing the individuals into training and test sets in a 4:1 ratio. This process was repeated 100 times. The optimal hyperparameter (α, regularization strength) was selected by conducting a four-fold cross-validation on the training set. The model performance was evaluated on the test set using Pearson’s correlation between the empirical and predicted PANSS scores. In accordance with previous work [50,51], we implemented the NeuroComBat harmonization method to corrected for multi-site effects in the training and test sets, respectively. All models were built and evaluated using the scikit-learn library (version: 1.3.1) [74] in Python (version: 3.10.12).

## 3. Results

A total of 209 SZ patients and 270 healthy controls from 8 different acquisition sites were included in this study. The handedness of all participants is predominantly righthanded. There are no significant differences in gender and age between the SZ and HC groups (gender: female/male = 65/144 for SZ, female/male = 91/179 for HC, Chi-squared test, $\chi^{2}=0.25$, p=0.61; age: 36.50±11.02 years old for SZ, 36.10±10.67 years old for HC, Student’s *t*-test, t=0.40, p=0.69). There are also no significant differences observed in the mean motion of resting-state fMRI data between the SZ and HC groups (mean FD: 0.17 ± 0.07 mm for SZ, 0.16 ± 0.07 mm for HC, Student’s *t*-test, t=1.37, p=0.17). The detailed demographic information for each site is presented in Supplementary Table S1.

### 3.1. Functional Gradients and Their Hemispheric Asymmetry

**Figure 1:**
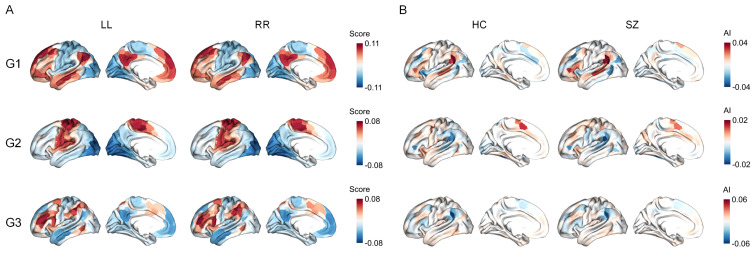
Asymmetry of intra-hemispheric functional gradients. (**A**) The mean spatial patterns of the first three intra-hemispheric gradients (G1, G2, and G3) across all participants. LL indicates the mean spatial patterns of gradients corresponding to the functional connectome within the left hemisphere. RR indicates the mean spatial patterns of gradients corresponding to the functional connectome within the right hemisphere. (**B**) Average AI (asymmetry index) values of intra-hemispheric functional gradients along the first three gradients for the healthy control (HC) and schizophrenia (SZ) groups. A positive AI value indicates leftward asymmetry, whereas a negative AI value indicates rightward asymmetry.

Through the diffusion map embedding algorithm, we derived the first three intra-hemispheric and inter-hemispheric functional gradient templates (G1, G2, and G3), respectively. The three gradients accounted for approximately 54.55% of the total variance in the average intra-hemispheric connectome, and approximately 52.93% of the total variance in the average inter-hemispheric connectome (Supplementary Figure S1B,C). Supplementary Figure S1A illustrates the spatial patterns of the first three intra-hemispheric and inter-hemispheric gradient templates. In line with previous literature [37,50], the principle gradient (G1) of both intra-hemispheric and inter-hemispheric connectomes captures a unimodal-to-transmodal hierarchical organization. The second gradient (G2) delineates a hierarchy axis where visual and somatomotor regions were situated at opposite ends. The third gradient (G3) reveals a hierarchy transitioning from somatomotor/default mode systems to multiple demand systems. As shown, the spatial patterns were highly similar between intra-hemispheric and inter-hemispheric gradient templates (Pearson’s r = 0.980 for G1, Pearson’s r = 0.987 for G2, Pearson’s r = 0.990 for G3) (Supplementary Figure S2). Figure 1A displayed the mean spatial patterns of the first three intra-hemispheric gradients across all participants, which were highly similar to the template gradients. The mean spatial patterns of the first three inter-hemispheric gradients across all participants were shown in Supplementary Figure S3A.

Despite the overall high degree of concordance, some differences were observed between the two hemispheres in both the SZ and HC groups. We assessed these differences or asymmetries using AI scores, which characterized differences in the position of homologous regions along functional gradients. Figure 1B showed the group-average AI maps of intra-hemispheric gradients for both SZ and HC groups, and the group-average AI maps of inter-hemispheric gradients were presented in Supplementary Figure S3B. We also showed Cohen’s d maps for intra-hemispheric and inter-hemispheric gradients (Supplementary Figure S3C,D), with FDR corrections to assess the statistical significance of AI values ($p_{FDR}<0.05$). We observed that hemispheric gradient asymmetries were prevalent in each of the first three gradients. For instance, for intra-hemispheric G1, regions with significant leftward asymmetry (positive AI values) were located in the inferior parietal cortex, lateral temporal lobe, and inferior frontal gyrus. Conversely, regions with significant rightward asymmetry (negative AI values) were observed in the insula, middle frontal gyrus, and cingulate.

### 3.2. Network-Level Comparisons

**Figure 2:**
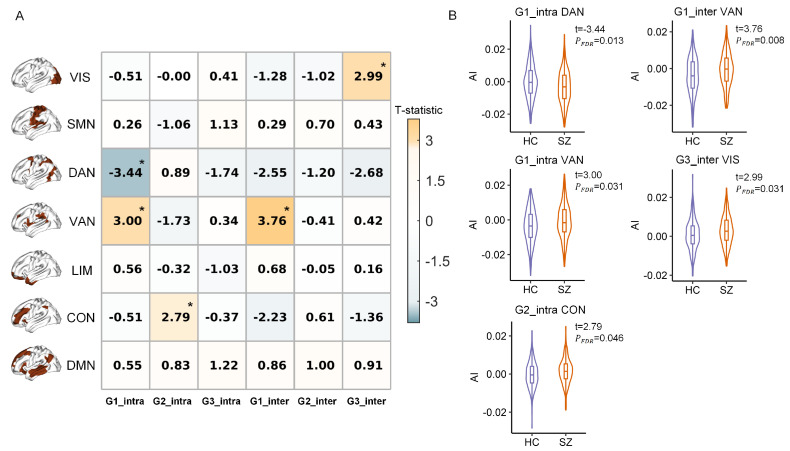
Network-level comparisons of hemispheric functional gradient asymmetry between healthy controls (HC) and patients with schizophrenia (SZ). (**A**) The Student’s *t* of the SZ-HC differences in hemispheric gradient asymmetry for each resting-state functional network, with statistical significance set at FDR-corrected *p* < 0.05. A positive Student’s *t* indicates a higher asymmetry index in the SZ group compared to the HC group. * Denotes significant between-group differences. (**B**) The violin plot combining with boxplot for each significant between-group comparison in hemispheric gradient asymmetry. G1_intra, intra-hemispheric asymmetry of the first gradient; G1_inter, inter-hemispheric asymmetry of the first gradient. Resting-state networks: VIS, visual; SMN, somatomotor; DAN, dorsal attention; VAN, ventral attention; LIM, limbic; CON, control; DMN, default mode.

In this section, we investigated whether patients with SZ were associated with changes in the hemispheric gradient asymmetry of specific functional networks. We aggregated gradient scores within seven functional networks and calculated intra-hemispheric and inter-hemispheric AI values. Network-level analyses showed that, compared with healthy controls, patients with SZ exhibited significant alterations in hemispheric gradient asymmetries across multiple networks, including the dorsal attention, ventral attention, control, and visual networks (Figure 2A). More specifically, for intra-hemispheric G1, patients with SZ showed significantly increased rightward asymmetry in the dorsal attention network ($t=-3.44,p_{FDR}=0.013$), and an increased leftward-asymmetric trend in the ventral attention network ($t=3.00,p_{FDR}=0.031$). For intra-hemispheric G2, patients with SZ exhibited significantly increased leftward asymmetry in the control network ($t=2.79,p_{FDR}=0.046$). For inter-hemispheric G1, patients were associated with significantly reduced right hemisphere dominance in the ventral attention network ($t=3.76,p_{FDR}=0.008$). For inter-hemispheric G3, patients exhibited significantly improved leftward asymmetry in the visual network ($t=2.99,p_{FDR}=0.031$) (Figure 2B). The details of network-level comparisons are reported in Supplementary Table S2.

### 3.3. Region-Level Comparisons

**Figure 3:**
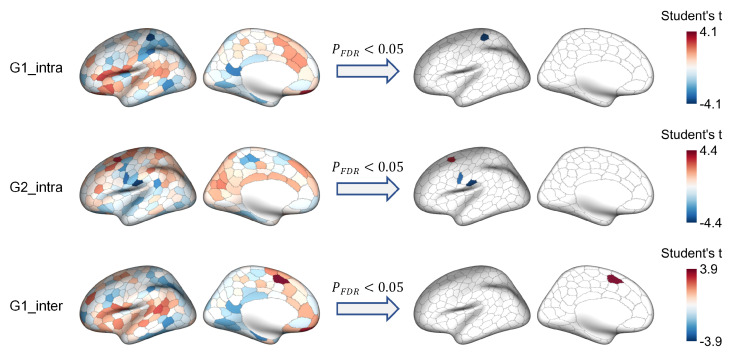
Significant region-level comparisons of hemispheric functional gradient asymmetry between healthy controls (HC) and patients with schizophrenia (SZ). A positive Student’s *t* indicates a higher asymmetry index in the SZ group compared to the HC group.

We subsequently examined between-group differences in the hemispheric gradient asymmetry across the first three functional gradients at the regional level. We observed significant case-control differences in regional AI values along the first and second gradients (G1 and G2) (Figure 3). Specifically, for intra-hemispheric G1, patients with SZ exhibited significantly decreased AI values in a parcel (SomMot_16, $t=-4.10,p_{FDR}=0.010$), which is located in the postcentral gyrus. For intra-hemispheric G2, patients with SZ showed significant abnormal hemispheric gradient asymmetries in three parcels, including increased AI values in a parcel in the dorsal prefrontal cortex (Cont_PFCd_1, $t=3.42,p_{FDR}=0.045$), and reduced AI values in two parcels in the somatomotor regions (SomMot_25, $t=-3.44,p_{FDR}=0.045$; SomMot_28, $t=-4.39,p_{FDR}=0.003$). For inter-hemispheric G1, patients with SZ had significantly higher AI values in a parcel in the medial superior frontal gyrus (SalVentAttn_FrMed_2, $t=3.89,p_{FDR}=0.023$). We detected no significant between-group differences in hemispheric gradient asymmetry for the third gradient as well as inter-hemispheric G2 (Supplementary Figure S4). More details regarding the regional differences in gradient asymmetries are provided in Supplementary Table S3.

### 3.4. Relation to Clinical Data

**Figure 4:**
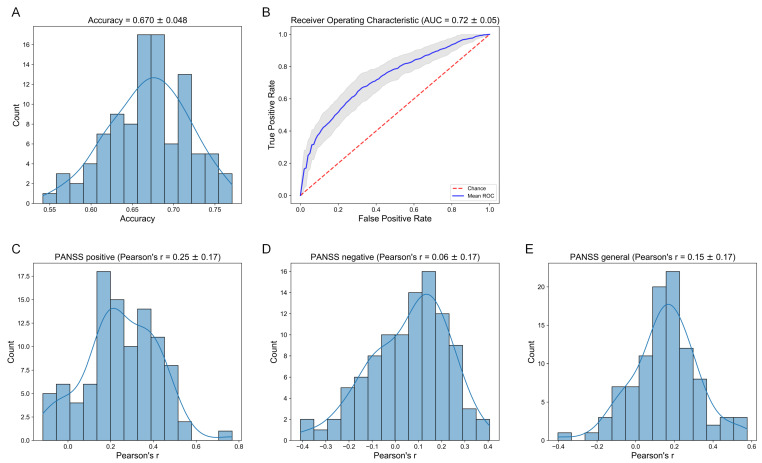
Relation to clinical data. (**A**) The distribution of classification accuracy across 100 random sample splits. (**B**) The mean ROC curve across 100 random sample splits. The red line indicates the baseline of the classification model. The grey shaded area surrounding the mean ROC curve (blue curve) represents the confidence interval of the mean ROC curve. (**C**) The distribution of Pearson correlation between empirical and predicted PANSS positive scores on the test set across 100 random sample splits. (**D**) The distribution of Pearson correlation between empirical and predicted PANSS negative scores on the test set across 100 random sample splits. (**E**) The distribution of Pearson correlation between empirical and predicted PANSS general psychopathology scores on the test set across 100 random sample splits.

Finally, we examined whether hemispheric gradient asymmetries could inform clinical measures. We selected gradient asymmetries (AI scores) corresponding to networks and regions with significant case-control differences, ultimately yielding 10 features. First, we trained a classification model using logistic regression with L2 penalty to evaluate whether gradient asymmetries could serve as a biomarker to distinguish healthy controls from SZ patients. We found that across 100 random splits, the classification accuracy on test sets was 0.670±0.048 (mean ± SD) (Figure 4A) and the AUC value was 0.72±0.05 (mean ± SD) (Figure 4B). These results highlighted that these gradient asymmetry features could be regarded as biomarkers for the diagnosis of schizophrenia. Then, we trained a ridge regression model to assess whether gradient asymmetries could predict PANSS scores. Figure 4C–E showed the distribution of prediction performance on test sets across 100 random splits. Out-of-sample predictions indicated an accuracy Pearson’s r of 0.25±0.17 for PANSS positive scores, a bad performance (Pearson’s r = 0.06±0.17) for PANSS negative scores, and a relatively weak accuracy (Pearson’s r = 0.15±0.17) for PANSS general psychopathology scores.

## 4. Discussion

In the present study, we capitalized on a gradient mapping framework to investigate the hemispheric asymmetry of functional connectome hierarchy in patients with SZ. We divided the hemispheric gradient asymmetry into two complementary components, intra- and inter-hemispheric asymmetry, which, respectively, reflected the lateralization in information specialization within hemispheres and information integration between hemispheres [48,52]. By harmonizing resting-state fMRI data from a large multi-site sample, we found that, compared to healthy controls, patients with SZ exhibited significantly altered intra-hemispheric asymmetries in the dorsal attention, ventral attention, and control networks, as well as altered inter-hemispheric asymmetries in the visual and ventral attention networks. Regional analyses revealed that patients with SZ showed abnormalities in hemispheric gradient asymmetries in several cortical regions in the somatomotor regions, dorsal prefrontal cortex, and medial superior frontal gyrus. Finally, by machine learning, we found that hemispheric asymmetry values with significant between-group differences could differentiate between patients and healthy controls with high accuracy and moderately predict PANSS positive scores. In sum, our findings highlight altered functional hierarchy asymmetry in patients with SZ, contributing to a deeper understanding of atypical brain lateralization in SZ.

Prior studies have shown that SZ is associated with a multifaceted disruption in functional hierarchy, including contracted unimodal-to-transmodal [45], visual-to-sensorimotor [75], and cerebellar gradients [46], as well as an expanded thalamic functional organization [47]. Here, we extend these findings by systematically evaluating SZ-related alterations in the hemispheric asymmetry of functional hierarchy. To the best of our knowledge, this is the first attempt to explore the abnormal brain lateralization in SZ patients using the concept of functional gradient. By conducting network-level analyses, we observed that patients exhibited abnormal hemispheric gradient asymmetries in the ventral attention, dorsal attention, control, and visual networks. Prior studies have indicated that patients with SZ showed disrupted functional hierarchy in these networks. For example, patients with SZ have significantly reduced within-network gradient dispersion in the dorsal attention, visual, and control networks along the visual-somatomotor axis [75]. Patients with drug-naïve first-episode SZ show higher gradient scores in the ventral attention network along the unimodal-to-transmodal axis [76]. Our findings further reveal a disturbance in the lateralization of functional hierarchy within these networks, indicating that SZ might be associated with multi-system impairments in brain lateralized information processing. Abnormal network-level hemispheric asymmetries are found in multiple different functional gradients, suggesting that the effects of SZ on hemispheric specialization manifest across multiple hierarchical organizations reflecting systematic shifts in distinct functional domains. These multi-axis alterations in SZ might be implicated with sophisticated symptoms. For instance, abnormal lateralization in the visual-somatomotor axis may potentially correspond to perceptual deficits [77]. Additionally, we observe that the ventral attention network exhibits the largest effect size for between-group differences and is involved in both intra- and inter-hemispheric asymmetry alterations. The ventral attention network is responsible for detecting salient stimuli and recruiting relevant functional networks [78], and is considered to play a crucial role in the pathogenesis of SZ [79]. Our findings support the pathological relevance of the ventral attention network in SZ and suggest altered lateralization of information processing in the ventral attention network for patients with SZ.

By assessing between-group differences in regional hemispheric asymmetries across the first three gradients, we observed SZ-related alterations in lateralization in several cortical regions, involving the precentral gyrus, postcentral gyrus, dorsal prefrontal cortex, and medial frontal gyrus. The precentral gyrus serves as the site of the primary motor cortex and is associated with regulating voluntary motor movements [80]. The postcentral gyrus, comprising the primary somatosensory cortex, is accountable for perceiving diverse somatic sensations originated from the body [81]. A substantial body of research has consistently demonstrated that patients with SZ have aberrant functional connectivity in the precentral and postcentral gyrus, indicating these regions are intimately associated with the pathology of SZ [82,83]. Altered lateralization in the precentral and postcentral regions might be associated with somatosensory and motor dysfunctions in SZ [84,85]. The dorsal prefrontal cortex plays an important role in executive functions, such as working memory and inhibition [86,87]. Previous studies have reported neuronal pathology [88] and disrupted cortical parallel circuits [89] in the dorsal prefrontal cortex for patients with SZ. The medial frontal gyrus is related to executive functions [90] and has been reported to exhibit reduced gray matter volume in patients with SZ [91]. We speculate that abnormal hemispheric asymmetry in the dorsal prefrontal and medial frontal cortices might correspond to executive function deficits in patients with SZ [92]. However, due to the dearth of detained clinical data, we are unable to establish direct associations between patients’ symptoms and altered hemispheric asymmetry of functional hierarchy in these regions. In addition, these identified regions exhibit an overlap with findings from prior hemispheric studies regarding SZ. More specifically, previous studies have reported that patients with SZ exhibit abnormal functional connectivity asymmetry in the frontal lobe [32], significant alterations in lateralization of resting-state fMRI variability in the postcentral and precentral areas [31], a trend toward decreased hemispheric asymmetry in nodal efficiency of the brain anatomical network in the medial superior frontal gyrus [93], and significantly reduced homotopic inter-hemispheric functional connectivity and voxel-mirrored homotopic connectivity in the postcentral and precentral regions [94,95]. We find that hemispheric asymmetries of functional hierarchy with significant case-control differences are relevant to clinical measures, with classifying patients and healthy individuals with high accuracy, as well as predicting PANSS positive scores. Prior studies have demonstrated that hemispheric asymmetries of functional hierarchy can predict autistic traits in autism [50] and depression traits in major depressive disorder [51]. Our findings emphasize the potential of functional hierarchy asymmetries for the clinical diagnosis and application of SZ.

There are several limitations to our findings that warrant consideration. First, the large sample we analyzed is aggregated from multiple sites with varying acquisition parameters. While we applied the NeuroCombat method to adjust for site effects, further work should employ a large sample collected under consistent parameters to validate our findings. Second, the interpretation of our findings should be restricted to participants whose dominant hand are right-handed. Given that handedness can influence the asymmetries of cerebral cortex [96], future studies can further expand our findings on left-handed participants. Third, due to the limited availability of detailed information on illness duration for many patients, we are unable to assess the impact of the duration of illness and disease stage on our findings. Further research aimed at establishing the association between disease duration and stage and functional gradient asymmetry would be of great significance.

## 5. Conclusions

The current study provides evidence that schizophrenia is associated with abnormal hemispheric asymmetry in functional hierarchy. In particular, the most pronounced abnormalities in gradient asymmetry are observed in the ventral attention network, highlighting the importance of targeting the ventral attention system for neuromodulation treatments of schizophrenia and for advancing our understanding of its pathology. Furthermore, these functional hierarchy asymmetries are capable of distinguishing between patients and healthy controls and moderately predict PANSS positive scores, indicating the potential of functional hierarchy asymmetry in diagnosing schizophrenia. Altogether, this study offers novel insights into the disrupted brain lateralization in schizophrenia and has significant implications for the development of new biomarkers for the diagnosis, intervention, and therapy of schizophrenia.

## Data Availability

The datasets were obtained from COINS (http://coins.mrn.org/dx, accessed on 12 February 2023), the OpenNeuro database (https://openneuro.org/, accessed on 20 August 2024), the DecNef Project Brain Data Repository (https://bicr-resource.atr.jp/srpbsopen/, accessed on 10 January 2023), and the Human Connectome Project (HCP) database (https://www.humanconnectome.org/study/hcp-young-adult, accessed on 20 December 2024).

## Supplementary Materials

The following supporting information can be downloaded at: https://www.mdpi.com/article/10.3390/brainsci15030313/s1, Figure S1: The template gradients. (A) The spatial patterns of the first three intra-hemispheric template gradients and the first three inter-hemispheric template gradients. (B) The variance in the average intra-hemispheric functional connectome explained by the first 10 gradient components. (C) The variance in the average inter-hemispheric functional connectome explained by the first 10 gradient components; Figure S2: The spatial correlations between intra-hemispheric and inter-hemispheric gradient templates; Figure S3: Asymmetry of hemispheric functional gradients. (A) The mean spatial patterns of the first three inter-hemispheric gradients (G1, G2, and G3) across all participants. LR indicates the mean spatial patterns of gradients corresponding to the LR connectome. RL indicates the mean spatial patterns of gradients corresponding to the RL connectome. (B) Average AI (asymmetry index) values of inter-hemispheric functional gradients along the first three gradients for the healthy control (HC) and schizophrenia (SZ) groups. A positive AI value indicates leftward asymmetry, whereas a negative AI value indicates rightward asymmetry. (C) Cohen’s d maps of intra-hemispheric AI values for the HC and SZ groups. (D) Cohen’s d maps of inter-hemispheric AI values for the HC and SZ groups; Figure S4: Region-level comparisons of hemispheric functional gradient asymmetry between healthy controls (HC) and patients with schizophrenia (SZ) for intra-hemispheric G3, inter-hemispheric G2, and inter-hemispheric G3. A positive Student’s *t* indicates a higher asymmetry index in the SZ group compared to the HC group; Table S1: Demographic characteristics of participants per site; Table S2: Network-level between-group differences in hemispheric gradient asymmetry; Table S3: Region-level between-group differences in hemispheric gradient asymmetry. References [97,98,99,100,101,102,103,104,105,106,107,108,109,110,111,112,113,114,115,116,117] are cited in the supplementary materials.
