# Supplementary material for: Altered Hemispheric Asymmetry of Functional Hierarchy in Schizophrenia
Source: Brain Sci. 2025 Mar 16;15(3):313. doi: 10.3390/brainsci15030313 (PMC11940334; doi:10.3390/brainsci15030313)
Supplement: Supplementary file 1 [file brainsci-15-00313-s001.zip › Supplementary material.pdf]

Supplementary methods

Participants and Data acquisition

COBRE dataset[1]: This open-access dataset was collected at the Mind Research Network. This collection was approved by the institutional review board (IRB) of the University of New Mexico (UNM) and all subjects provided written informed consent. Each participant was diagnosed by the Structured Clinical Interview for DSM-IV Axis I Disorders[2]. The symptom ratings of patients were assessed through the Positive and Negative Syndrome Scale (PANSS)[3]. All imaging data were acquired on a Siemens 3 T TIM Trio scanner, using a 12-channel head coil. The T1-weighted images were acquired with a multi-echo MPRAGE sequence (TE = 1.64, 3.5, 5.36, 7.22, 9.08ms, TR = 2.53s, TI = 1.2s, flip angle = 7°, slice thickness = 1mm, FOV = 256mm, resolution = 256×256, voxel size = 1.0×1.0×1.0mm). The fMRI data were acquired with a gradient-echo echoplanar pulse sequence (TE = 29ms, TR = 2s, flip angle = 75°, FOV = 240mm, 33 slices, matrix size = 64×64, voxel size = 3.75×3.75×4.55mm).

BrainGluSchi dataset[4]: This study was approved by the UNM Institutional Review Board and subjects gave written informed consent. Participants were diagnosed by the DSM-IV-TR[5]. Imaging data were collected on a 3 Tesla scanner (VB-17; 12 channel head-coil). The T1-weighted images were acquired with 3D-MPRAGE (TE = 3.87ms, TR = 1.5s, TI = 700ms, flip angle = 10°, slice thickness = 1mm, FOV = 256×256mm). The fMRI data were collected using the following parameters: TE = 29ms, TR = 2s, flip angle = 75°, FOV = 240mm, 33 slices, voxel size = 3.8×3.8×3.5mm.

UCLA-CNP dataset[6]: Subjects gave written informed consent in accordance with procedures approved by the Institutional Review Boards at UCLA and the Los Angeles County Department of Mental Health. Participants were diagnosed using the Structured Clinical Interview for DSM-IV[5]. The MRI data were collected on one of two 3T Siemens Trio scanners. The T1-weighted MPRAGE images were acquired with the following parameters: TR = 1.9s, TE = 2.26ms, FOV = 250mm, matrix = 256×256, 176 slices, slice thickness = 1mm. The fMRI data were collected with a $T2^*$-weighted echoplanar imaging (EPI) sequence (TR = 2s, TE = 30ms, matrix = 64×64, flip angle = 90°, slice thickness = 4mm, 34 slices, FOV = 192mm).

SRPBS database[7] (SWA, UTO, KUT, KTT): All participants provided written informed consent. The recruitment procedures and experimental protocols for the SWA\UTO\KUT\KTT dataset were approved

by the institutional review boards of the Showa University\University of Tokyo Faculty of Medicine\Kyoto University\Kyoto University. Subjects were diagnosed using the DSM-IV-TR[5] or DSM-5[8]. For the SWA dataset, the imaging data were collected using a 3T Siemens Verio scanner. The T1-weighted images were acquired with the following parameters: TR = 2.3s, TE = 2.98ms, TI = 900ms, flip angle = 9o, FOV = 256mm, matrix = 256×256, voxel size = 1×1×1mm; the fMRI data were collected with the following parameters: TR = 2.5s, TE = 30ms, flip angle = 80°, FOV = 212mm, matrix = 64×64, 40 slices, in-plane resolution = 3.3×3.3mm, slice thickness = 3.2mm. For the UTO dataset, the imaging data were collected using a 3T GE MR750w scanner. The T1-weighted images were acquired with the following parameters: TR = 7.7ms, TE = 3.1ms, TI = 400ms, flip angle = 8°, FOV = 240mm, matrix = 256×256, voxel size = 1×1×1.2mm; the fMRI data were collected with the following parameters: TR = 2.5s, TE = 30ms, flip angle = 80°, FOV = 212mm, matrix = 64×64, 40 slices, in-plane resolution = 3.3×3.3mm, slice thickness = 3.2mm. For the KUT dataset, the imaging data were collected using a 3T Siemens TimTrio scanner. The T1-weighted images were acquired with the following parameters: TR = 2s, TE = 3.4ms, TI = 990ms, flip angle = 8°, FOV = 225×240mm, matrix = 240×256, voxel size = 0.9375×0.9375×1.0mm; the fMRI data were collected with the following parameters: TR = 2.5s, TE = 30ms, flip angle = 80°, FOV = 212mm, matrix = 64 × 64, 40 slices, in-plane resolution = 3.3125×3.3125mm, slice thickness = 3.2mm. For the KTT dataset, the imaging data were collected using a 3T Siemens Trio scanner. The T1-weighted images were acquired with the following parameters: TR = 2s, TE = 4.38ms, TI = 990ms, flip angle = 8°, FOV = 225×240mm, matrix = 240×256, voxel size = 0.9375×0.9375×1.0mm; the fMRI data were collected with the following parameters: TR = 2s, TE = 30ms, flip angle = 90°, FOV = 256×192mm, matrix = 64×48, 30 slices, in-plane resolution = 4×4mm, slice thickness = 4mm.

The Neural Basis of Visual Shape Completion in Schizophrenia and Bipolar Disorder dataset[9-10]: This dataset was downloaded from the openneuro[11] platform (ds005073 and ds003404). All participants provided written informed consent. The study was approved by the Rutgers University Institutional Review Board. All participants were diagnosed using the DSM-5[8]. The imaging data were collected on a Siemens Tim Trio scanner. The T1-weighted imaging data were collected using the following parameters: TR = 2.4s, TE = 2.31ms, TI = 1s, slice thickness = 0.8mm, flip angle = 8°; the multiband EPI data were collected using a 32-channel head coil with the following parameters: TR = 785ms, TE =

34.8ms, flip angle = 55°, FOV = 211mm, 60 slices, 2.4mm isotropic voxels, multiband acceleration factor = 6.

Due to the extreme imbalance between the number of healthy controls (HC) and participants with schizophrenia (SZ) in five datasets (UCLA-CNP, SWA, UTO, KUT, and KTT), we performed the optimal group matching using the R package MatchIt[32].

Data preprocessing

Results included in this manuscript come from preprocessing performed using fMRIPrep 20.2.3[12-13], which is based on Nipype 1.6.1[14-15].

Anatomical data preprocessing

A total of 1 T1-weighted (T1w) images were found within the input BIDS dataset. The T1-weighted (T1w) image was corrected for intensity non-uniformity (INU) with N4BiasFieldCorrection[16], distributed with ANTs 2.3.3[17], and used as T1w-reference throughout the workflow. The T1w-reference was then skull-stripped with a Nipype implementation of the antsBrainExtraction.sh workflow (from ANTs), using OASIS30ANTs as target template. Brain tissue segmentation of cerebrospinal fluid (CSF), white-matter (WM) and gray-matter (GM) was performed on the brain-extracted T1w using fast[18] (FSL 5.0.9). Brain surfaces were reconstructed using recon-all[19] (FreeSurfer 6.0.1), and the brain mask estimated previously was refined with a custom variation of the method to reconcile ANTs-derived and FreeSurfer-derived segmentations of the cortical gray-matter of Mindboggle[20]. Volume-based spatial normalization to two standard spaces (MNI152NLin6Asym, MNI152NLin2009cAsym) was performed through nonlinear registration with antsRegistration (ANTs 2.3.3), using brain-extracted versions of both T1w reference and the T1w template. The following templates were selected for spatial normalization: FSL's MNI ICBM 152 non-linear 6th Generation Asymmetric Average Brain Stereotaxic Registration Model[21] [TemplateFlow ID: MNI152NLin6Asym], ICBM 152 Nonlinear Asymmetrical template version 2009c[22] [TemplateFlow ID: MNI152NLin2009cAsym],

Functional data preprocessing

For each of the 1 BOLD run found per subject (across all tasks and sessions), the following preprocessing

was performed. First, a reference volume and its skull-stripped version were generated using a custom methodology of fMRIPrep. Susceptibility distortion correction (SDC) was omitted. The BOLD reference was then co-registered to the T1w reference using bbregister (FreeSurfer) which implements boundary-based registration[23]. Co-registration was configured with six degrees of freedom. Head-motion parameters with respect to the BOLD reference (transformation matrices, and six corresponding rotation and translation parameters) are estimated before any spatiotemporal filtering using mcflirt[24] (FSL 5.0.9). BOLD runs were slice-time corrected using 3dTshift from AFNI 20160207[25]. The BOLD time-series were resampled onto the following surfaces (FreeSurfer reconstruction nomenclature): fsaverage5, fsaverage. The BOLD time-series (including slice-timing correction when applied) were resampled onto their original, native space by applying the transforms to correct for head-motion. These resampled BOLD time-series will be referred to as preprocessed BOLD in original space, or just preprocessed BOLD. The BOLD time-series were resampled into standard space, generating a preprocessed BOLD run in MNI152NLin6Asym space. First, a reference volume and its skull-stripped version were generated using a custom methodology of fMRIPrep. Grayordinates files[26] containing 91k samples were also generated using the highest-resolution fsaverage as intermediate standardized surface space. Several confounding time-series were calculated based on the preprocessed BOLD: framewise displacement (FD), DVARS and three region-wise global signals. FD was computed using two formulations following Power[27] (absolute sum of relative motions) and Jenkinson[24] (relative root mean square displacement between affines). FD and DVARS are calculated for each functional run, both using their implementations in Nipype (following the definitions by Power et al.). The three global signals are extracted within the CSF, the WM, and the whole-brain masks. Additionally, a set of physiological regressors were extracted to allow for component-based noise correction[28] (CompCor). Principal components are estimated after high-pass filtering the preprocessed BOLD time-series (using a discrete cosine filter with 128s cut-off) for the two CompCor variants: temporal (tCompCor) and anatomical (aCompCor). tCompCor components are then calculated from the top 2% variable voxels within the brain mask. For aCompCor, three probabilistic masks (CSF, WM and combined CSF+WM) are generated in anatomical space. The implementation differs from that of Behzadi et al. in that instead of eroding the masks by 2 pixels on BOLD space, the aCompCor masks are subtracted a mask of pixels that likely contain a volume fraction of GM. This mask is obtained by dilating a GM mask extracted from the FreeSurfer's aseg segmentation, and it ensures components are not extracted from voxels containing a

minimal fraction of GM. Finally, these masks are resampled into BOLD space and binarized by thresholding at 0.99 (as in the original implementation). Components are also calculated separately within the WM and CSF masks. For each CompCor decomposition, the k components with the largest singular values are retained, such that the retained components' time series are sufficient to explain 50 percent of variance across the nuisance mask (CSF, WM, combined, or temporal). The remaining components are dropped from consideration. The head-motion estimates calculated in the correction step were also placed within the corresponding confounds file. The confound time series derived from head motion estimates and global signals were expanded with the inclusion of temporal derivatives and quadratic terms for each[29]. Frames that exceeded a threshold of 0.5 mm FD or 1.5 standardised DVARS were annotated as motion outliers. All resamplings can be performed with a single interpolation step by composing all the pertinent transformations (i.e. head-motion transform matrices, susceptibility distortion correction when available, and co-registrations to anatomical and output spaces). Gridded (volumetric) resamplings were performed using antsApplyTransforms (ANTs), configured with Lanczos interpolation to minimize the smoothing effects of other kernels[30]. Non-gridded (surface) resamplings were performed using mri_vol2surf (FreeSurfer).

Many internal operations of fMRIPrep use Nilearn 0.6.2[31], mostly within the functional processing workflow. For more details of the pipeline, see the section corresponding to workflows in fMRIPrep's documentation.

Copyright Waiver

The above boilerplate text was automatically generated by fMRIPrep with the express intention that users should copy and paste this text into their manuscripts unchanged. It is released under the CC0 license.

Supplementary figures

Figure S1. The template gradients. (A) The spatial patterns of the first three intra-hemispheric template gradients and the first three inter-hemispheric template gradients. (B). The variance in the average intra-hemispheric functional connectome explained by the first 10 gradient components. (C). The variance in the average inter-hemispheric functional connectome explained by the first 10 gradient components.

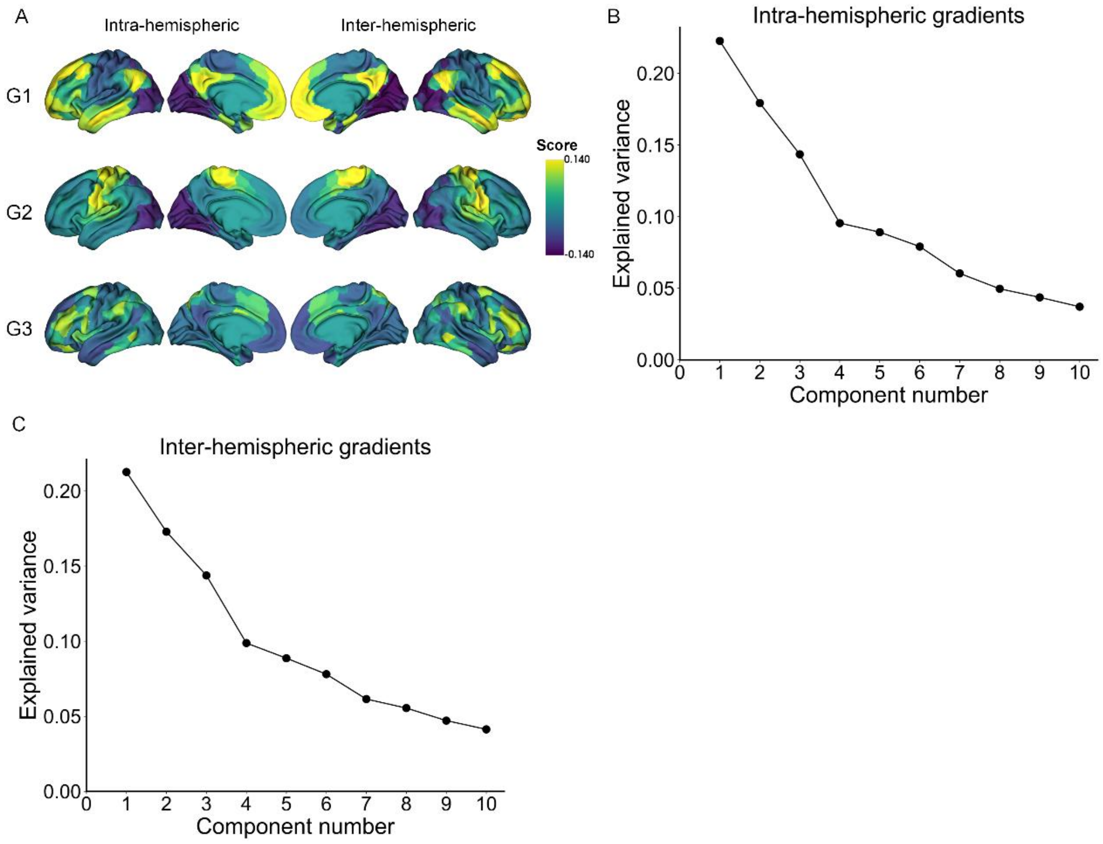

Figure S2. The spatial correlations between intra-hemispheric and inter-hemispheric gradient templates.

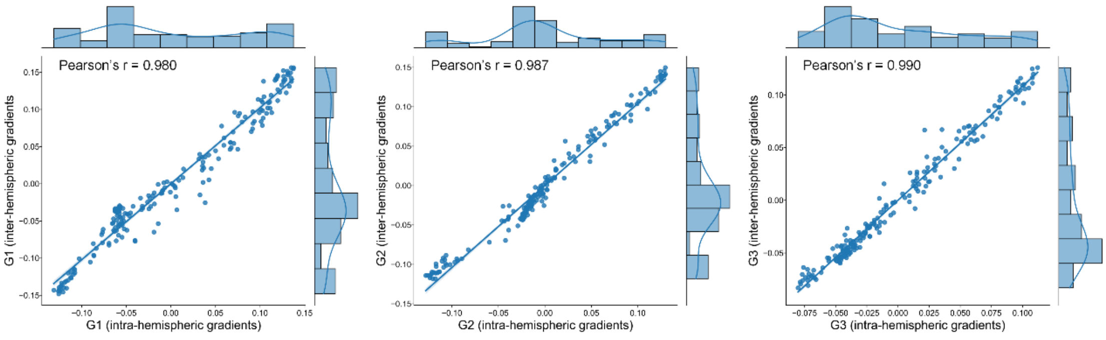

Figure S3. Asymmetry of hemispheric functional gradients. (A). The mean spatial patterns of the first three inter-hemispheric gradients (G1, G2, and G3) across all participants. LR indicates the mean spatial patterns of gradients corresponding to the LR connectome. RL indicates the mean spatial patterns of gradients corresponding to the RL connectome. (B). Average AI (asymmetry index) values of inter-hemispheric functional gradients along the first three gradients for the healthy control (HC) and schizophrenia (SZ) groups. A positive AI value indicates leftward asymmetry, whereas a negative AI value indicates rightward asymmetry. (C). Cohen's d maps of intra-hemispheric AI values for the HC and SZ groups. (D) Cohen's d maps of inter-hemispheric AI values for the HC and SZ groups.

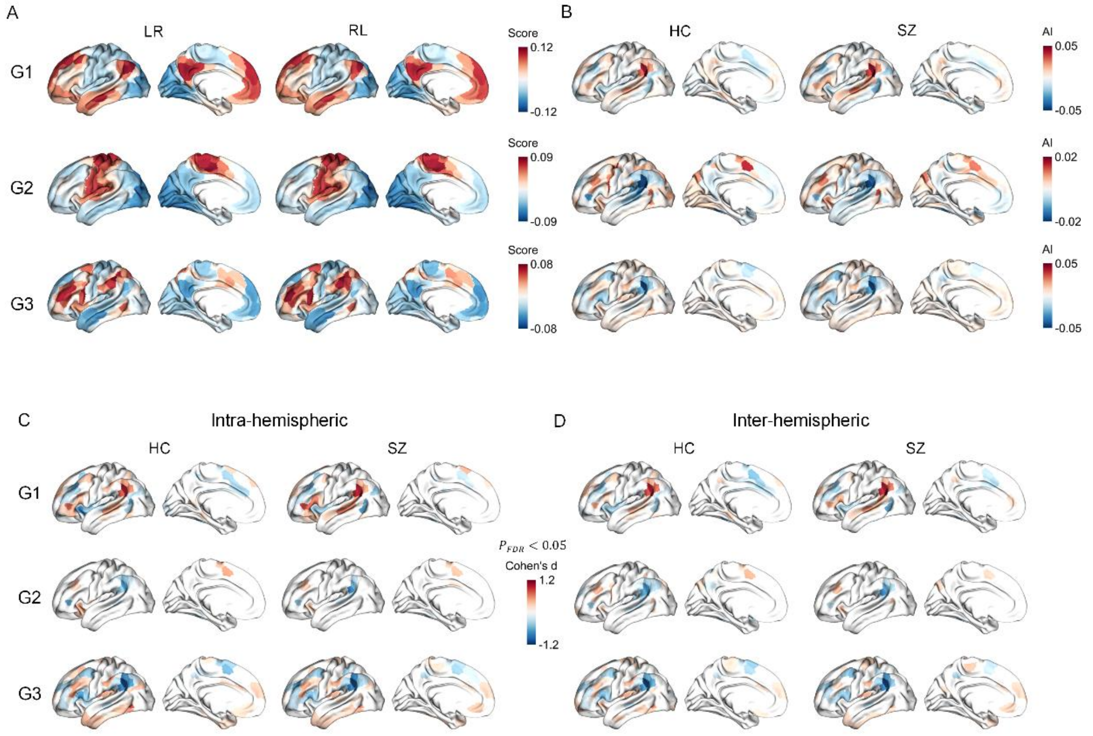

Figure S4. Region-level comparisons of hemispheric functional gradient asymmetry between healthy controls (HC) and patients with schizophrenia (SZ) for intra-hemispheric G3, inter-hemispheric G2, and inter-hemispheric G3. A positive Student's t indicates a higher asymmetry index in the SZ group compared to the HC group.

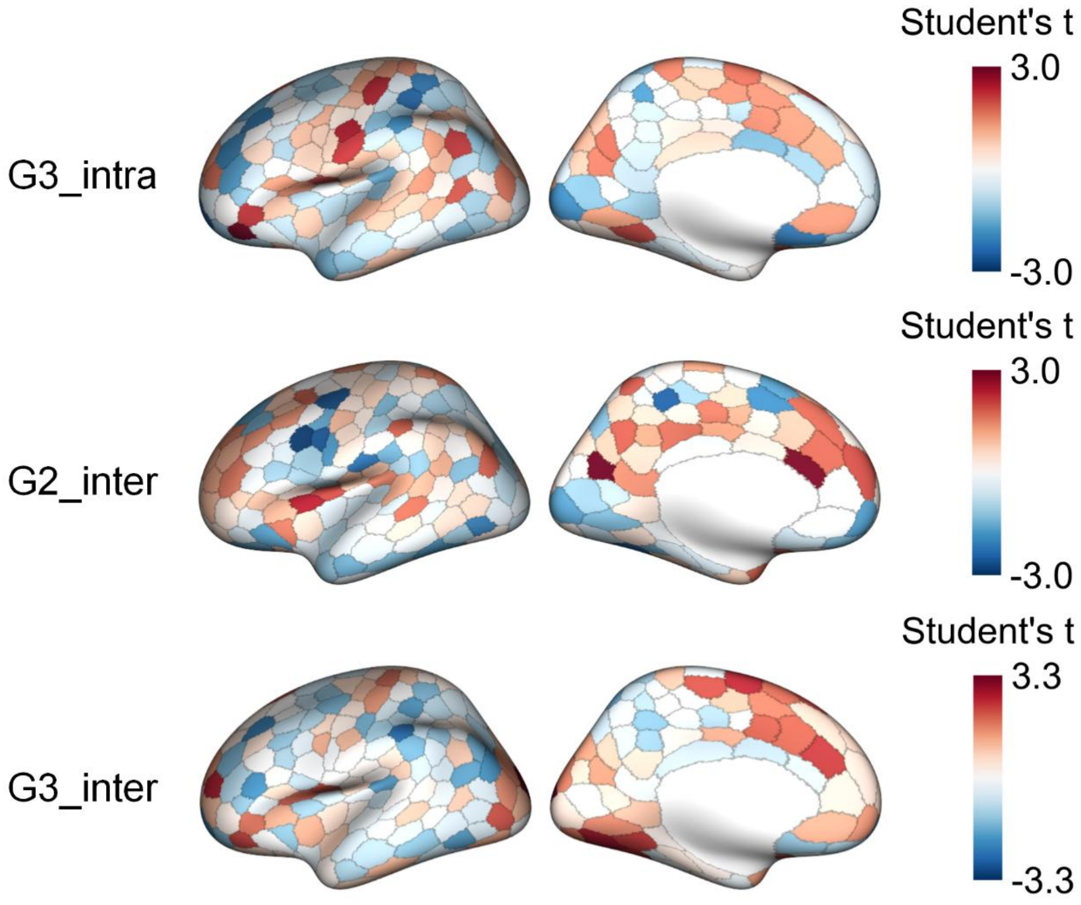

Reference

1. Aine, C. J., et al. "Multimodal neuroimaging in schizophrenia: description and dissemination." Neuroinformatics 15 (2017): 343-364.
2. First, Michael B. "Structured clinical interview for DSM-IV axis I disorders." Biometrics Research Department (1997).
3. Kay, Stanley R., Abraham Fiszbein, and Lewis A. Opler. "The positive and negative syndrome scale (PANSS) for schizophrenia." Schizophrenia bulletin 13.2 (1987): 261-276.
4. Bustillo, Juan R., et al. "Glutamatergic and neuronal dysfunction in gray and white matter: a spectroscopic imaging study in a large schizophrenia sample." Schizophrenia bulletin 43.3 (2017): 611-619.1
5. American Psychiatric Association A, American Psychiatric Association. Diagnostic and statistical manual of mental disorders: DSM-IV[M]. Washington, DC: American psychiatric association, 1994.
6. Poldrack, Russell A., et al. "A phenome-wide examination of neural and cognitive function." Scientific data 3.1 (2016): 1-12.
7. Tanaka S C, Yamashita A, Yahata N, et al. A multi-site, multi-disorder resting-state magnetic resonance image database[J]. Scientific data, 2021, 8(1): 227.
8. American Psychiatric Association, D. S. M. T. F., and D. S. American Psychiatric Association. Diagnostic and statistical manual of mental disorders: DSM-5. Vol. 5. No. 5. Washington, DC: American psychiatric association, 2013.
9. Keane, Brian P., et al. "Dorsal attention network activity during perceptual organization is distinct in schizophrenia and predictive of cognitive disorganization." European Journal of Neuroscience 57.3 (2023): 458-478.
10. Keane, Brian P., et al. "Brain network mechanisms of visual shape completion." NeuroImage 236 (2021): 118069.
11. Markiewicz, Christopher J., et al. "The OpenNeuro resource for sharing of neuroscience data." Elife 10 (2021): e71774.
12. Esteban, Oscar, Ross Blair, Christopher J. Markiewicz, Shoshana L. Berleant, Craig Moodie, Feilong Ma, Ayse Ilkay Isik, et al. 2018. “FMRIPrep.” Software. Zenodo.

https://doi.org/10.5281/zenodo.852659.

13. Esteban, Oscar, et al. "fMRIPrep: a robust preprocessing pipeline for functional MRI." Nature methods 16.1 (2019): 111-116.
14. Gorgolewski, Krzysztof, et al. "Nipype: a flexible, lightweight and extensible neuroimaging data processing framework in python." Frontiers in neuroinformatics 5 (2011): 12318.
15. Gorgolewski, Krzysztof J., Oscar Esteban, Christopher J. Markiewicz, Erik Ziegler, David Gage Ellis, Michael Philipp Notter, Dorota Jarecka, et al. 2018. “Nipype.” Software. Zenodo. https://doi.org/10.5281/zenodo.596855.
16. Tustison, Nicholas J., et al. "N4ITK: improved N3 bias correction." IEEE transactions on medical imaging 29.6 (2010): 1310-1320.
17. Avants, Brian B., et al. "Symmetric diffeomorphic image registration with cross-correlation: evaluating automated labeling of elderly and neurodegenerative brain." Medical image analysis 12.1 (2008): 26-41.
18. Zhang, Yongyue, Michael Brady, and Stephen Smith. "Segmentation of brain MR images through a hidden Markov random field model and the expectation-maximization algorithm." IEEE transactions on medical imaging 20.1 (2001): 45-57.
19. Dale, Anders M., Bruce Fischl, and Martin I. Sereno. "Cortical surface-based analysis: I. Segmentation and surface reconstruction." Neuroimage 9.2 (1999): 179-194.
20. Klein, Arno, et al. "Mindboggling morphometry of human brains." PLoS computational biology 13.2 (2017): e1005350.
21. Evans, Alan C., et al. "Brain templates and atlases." Neuroimage 62.2 (2012): 911-922.
22. Fonov, Vladimir S., et al. "Unbiased nonlinear average age-appropriate brain templates from birth to adulthood." NeuroImage 47 (2009): S102.
23. Greve, Douglas N., and Bruce Fischl. "Accurate and robust brain image alignment using boundary-based registration." Neuroimage 48.1 (2009): 63-72.
24. Jenkinson, Mark, et al. "Improved optimization for the robust and accurate linear registration and motion correction of brain images." Neuroimage 17.2 (2002): 825-841.
25. Cox, Robert W., and James S. Hyde. "Software tools for analysis and visualization of fMRI data." NMR in Biomedicine: An International Journal Devoted to the Development and

Application of Magnetic Resonance In Vivo 10.4-5 (1997): 171-178.

26. Glasser, Matthew F., et al. "The minimal preprocessing pipelines for the Human Connectome Project." Neuroimage 80 (2013): 105-124.
27. Power, Jonathan D., et al. "Methods to detect, characterize, and remove motion artifact in resting state fMRI." Neuroimage 84 (2014): 320-341.
28. Behzadi, Yashar, et al. "A component based noise correction method (CompCor) for BOLD and perfusion based fMRI." Neuroimage 37.1 (2007): 90-101.
29. Satterthwaite, Theodore D., et al. "An improved framework for confound regression and filtering for control of motion artifact in the preprocessing of resting-state functional connectivity data." Neuroimage 64 (2013): 240-256.
30. Lanczos, Cornelius. "Evaluation of noisy data." Journal of the Society for Industrial and Applied Mathematics, Series B: Numerical Analysis 1.1 (1964): 76-85.
31. Abraham, Alexandre, et al. "Machine learning for neuroimaging with scikit-learn." Frontiers in neuroinformatics 8 (2014): 71792.
32. Stuart E A, King G, Imai K, et al. MatchIt: nonparametric preprocessing for parametric causal inference[J]. Journal of statistical software, 2011.
